# Paravertebral catheter versus EPidural analgesia in Minimally invasive Esophageal resectioN: a randomized controlled multicenter trial (PEPMEN trial)

**DOI:** 10.1186/s12885-020-6585-1

**Published:** 2020-02-22

**Authors:** B. F. Kingma, W. J. Eshuis, E. M. de Groot, M. L. Feenstra, J. P. Ruurda, S. S. Gisbertz, W. ten Hoope, M. Marsman, J. Hermanides, M. W. Hollmann, C. J. Kalkman, M. D. P. Luyer, G. A. P. Nieuwenhuijzen, H. J. Scholten, M. Buise, M. J. van Det, E. A. Kouwenhoven, F. van der Meer, G. W. J. Frederix, E. Cheong, K. al Naimi, M. I. van Berge Henegouwen, R. van Hillegersberg

**Affiliations:** 1 Department of Surgery, University Medical Center Utrecht, POBOX 85500, 3508 GA Utrecht, The Netherlands; 2 Department of Surgery, Amsterdam UMC, Amsterdam, The Netherlands; 3 Department of Anesthesiology, Amsterdam UMC, Amsterdam, The Netherlands; 4 Department of Anesthesiology, University Medical Center Utrecht, Utrecht, The Netherlands; 5 Department of Surgery, Catharina Hospital, Eindhoven, The Netherlands; 6 Department of Anesthesiology, Catharina Hospital Eindhoven, Eindhoven, The Netherlands; 7 Department of Surgery, Hospital Group Twente, Almelo, The Netherlands; 8 Department of Anesthesiology, Hospital Group Twente Almelo, Almelo, The Netherlands; 9 Department of Public Health, Healthcare Innovation & Evaluation and Medical Humanities, University Medical Center Utrecht, Utrecht, the Netherlands; 10 Department of Surgery, Norfolk and Norwich University Hospital, Norwich, UK; 11 Department of Anesthesiology, Norfolk and Norwich University Hospital, Norwich, UK

## Abstract

**Background:**

Thoracic epidural analgesia is the standard postoperative pain management strategy in esophageal cancer surgery. However, paravertebral block analgesia may achieve comparable pain control while inducing less side effects, which may be beneficial for postoperative recovery. This study primarily aims to compare the postoperative quality of recovery between paravertebral catheter versus thoracic epidural analgesia in patients undergoing minimally invasive esophagectomy.

**Methods:**

This study represents a randomized controlled superiority trial. A total of 192 patients will be randomized in 4 Dutch high-volume centers for esophageal cancer surgery. Patients are eligible for inclusion if they are at least 18 years old, able to provide written informed consent and complete questionnaires in Dutch, scheduled to undergo minimally invasive esophagectomy with two-field lymphadenectomy and an intrathoracic anastomosis, and have no contra-indications to either epidural or paravertebral analgesia. The primary outcome is the quality of postoperative recovery, as measured by the Quality of Recovery-40 (QoR-40) questionnaire on the morning of postoperative day 3. Secondary outcomes include the QoR-40 questionnaire score Area Under the Curve on postoperative days 1–3, the integrated pain and systemic opioid score and patient satisfaction and pain experience according to the International Pain Outcomes (IPO) questionnaire, and cost-effectiveness. Furthermore, the groups will be compared regarding the need for additional rescue medication on postoperative days 0–3, technical failure of the pain treatment, duration of anesthesia, duration of surgery, total postoperative fluid administration day 0–3, postoperative vasopressor and inotrope use, length of urinary catheter use, length of hospital stay, postoperative complications, chronic pain at six months after surgery, and other adverse effects.

**Discussion:**

In this study, it is hypothesized that paravertebral analgesia achieves comparable pain control while causing less side-effects such as hypotension when compared to epidural analgesia, leading to shorter postoperative length of stay on a monitored ward and superior quality of recovery. If this hypothesis is confirmed, the results of this study can be used to update the relevant guidelines on postoperative pain management for patients undergoing minimally invasive esophagectomy.

**Trial registration:**

Netherlands Trial Registry, NL8037. Registered 19 September 2019.

## Background

Esophageal cancer is the 9th most common cancer worldwide and is increasingly diagnosed in the Western world, mainly due to the growing incidence of adenocarcinoma [1]. Esophagectomy is the core of curative treatment for esophageal cancer, achieving a 5-year survival rate of 40–50% when preceded by neoadjuvant therapy [2, 3]. Traditional open transthoracic esophagectomy is associated with substantial postoperative thoracic pain, mainly due to the large intercostal incision, which may lead to decreased mobility, (pulmonary) complications, and delayed recovery [4]. In this light, adequate pain management is essential during the early postoperative phase. Thoracic epidural analgesia is the current gold standard for pain control in this context, as it was shown to be superior to systemic opioids in terms of pain control and pulmonary complications after open esophagectomy [5, 6]. However, over the last decades, minimally invasive esophagectomy (MIE) is increasingly adopted, which is associated with less postoperative pain when compared to open surgery [7–9]. Furthermore, enhanced recovery protocols have been introduced for esophagectomy, aiming at fast mobilization and recovery after MIE [10–12]. As potential adverse effects of epidural analgesia include failed catheter placement, postoperative hypotension, and diminished mobilization, epidural analgesia may be counterproductive in achieving some of the key aims of enhanced recovery protocols for MIE [13, 14]. Moreover, epidural analgesia is associated with severe neurological complications such as epidural hematoma or abscess formation in up to 1 in 1000 patients, which should be considered a serious issue [15, 16]. Therefore, the value of epidural analgesia for MIE needs to be reconsidered in a state-of-the-art treatment setting.

In previous systematic reviews that included patients undergoing a variety of thoracotomy or thoracoscopy procedures (mostly lung surgery), paravertebral analgesia was found to achieve comparable pain relief while inducing less postoperative hypotension, urinary retention, and nausea when compared to epidural analgesia [17, 18]. While these results seem to represent rather solid evidence in favour of paravertebral analgesia for thoracic surgery in general, transthoracic esophagectomy includes an abdominal phase in addition to the thoracic procedure. This means that results from studies that primarily included patients undergoing only thoracic surgery may not be generalizable to the setting of transthoracic esophagectomy. The hypothesized advantages of paravertebral analgesia over epidural analgesia were previously explored in systematic reviews of patients undergoing esophagectomy, which revealed that paravertebral analgesia achieves comparable pain control and possibly causes less hypotensive events [19, 20]. However, both reviews highlighted the lack of high-quality prospective studies in patients undergoing MIE [19, 20].

Most available studies on the efficacy of paravertebral analgesia after esophagectomy are retrospective in nature and included patients who underwent resection by an open approach, implying a substantial risk of bias and the possibility that these results cannot be generalized to the setting of MIE. Therefore, the primary aim of the current study is to compare the postoperative quality of recovery between paravertebral and epidural analgesia in patients undergoing MIE. Secondary objectives are to compare the efficacy, side-effects, and cost-effectiveness between these analgesic modalities. The hypothesis of this study is that paravertebral analgesia achieves comparable pain control while causing less side-effects, leading to shorter length of stay on a monitored ward and superior postoperative quality of recovery compared to epidural analgesia.

## Methods

### Design

This study represents a multicenter randomized superiority controlled trial that is conducted in 4 Dutch high-volume centers for esophageal cancer surgery: (1) University Medical Center Utrecht, (2) Amsterdam UMC (3), Catharina Hospital Eindhoven, and (4) Hospital Group Twente Almelo. The current version of the study protocol (V1.2) was approved by the Medical Ethical Committee of the University Medical Center Utrecht (reference number 19–588) and was prospectively registered in the Netherlands Trial Registry (trial number NL8037). Amendments to the protocol will first be presented to the ethics board and the approved adjustments are then also processed in the Netherlands Trial Registry. As this study is considered to carry negligible risks for participating patients, a data monitoring committee (DMC) was judged to be redundant.

### Patient population

**Fig. 1:**
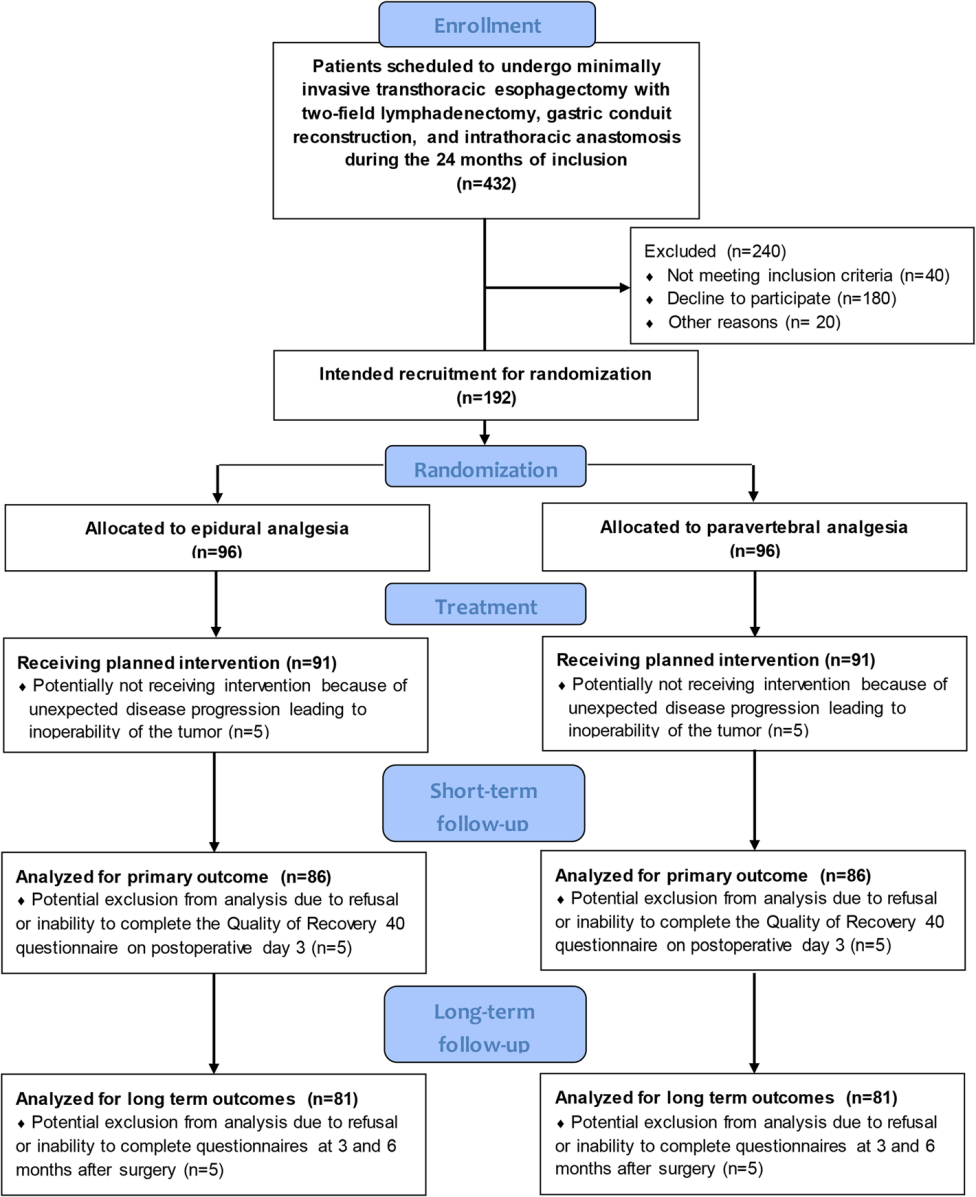
PEPMEN inclusion flowchart

Patients who are scheduled to undergo elective minimally invasive esophagectomy with two-field lymphadenectomy, gastric conduit reconstruction, and intrathoracic anastomosis (i.e. Ivor Lewis procedure) are eligible for participation and will be included according to the flowchart in Fig. 1. Exclusion criteria are severe comorbidity (ASA score > III), coagulation disorders that prohibit epidural analgesia according to the Dutch Society for Anesthesiology guidelines “Neuraxisblock and anti-coagulation”, other contraindications for epidural analgesia (e.g. local skin infection), allergy to local anesthetics, chronic opioid use prior to esophagectomy (> 3 months prior to the day of surgery), renal failure (eGFR < 50 mL/min.), inability to provide informed consent or complete questionnaires in Dutch, cervical lymph node dissection, and pregnancy.

### Allocation to the treatment arms

The coordinating investigators and involved trial nurses will screen patients for eligibility and obtain informed consent during patients’ visits to the preoperative outpatient clinic. After inclusion and ultimately the day before surgery, one of the coordinating investigators or trial nurses will enter the patient in Castor EDC, which is the primary data capturing platform for this study and allows digital randomization. Randomization will be performed in a 1:1 ratio and stratified per including center to minimize the risk of center bias. The coordinating investigators of the study will ensure that patients receive the allocated analgesic modality at the time of esophagectomy. As a sham epidural catheterization would need to be performed prior to the induction of general anesthesia in the paravertebral group, which is considered unethical, patients will not be blinded. Health care providers will also not be blinded, since blinding would likely increase the risk of errors in pain management to an unacceptable extent.

### Surgical technique

All patients will undergo MIE with two-field lymphadenectomy, gastric conduit reconstruction, and an intrathoracic anastomosis. The operation may be performed by conventional or robot-assisted thoracolaparoscopic surgery. The procedure starts with an abdominal laparoscopic phase, which involves mobilization of the stomach, abdominal lymphadenectomy and gastric conduit construction. Then, the patient will be placed in (semi) prone position for thoracoscopy to mobilize the esophagus and perform a mediastinal lymphadenectomy. One of the trocar ports is widened to a mini-thoracotomy for specimen extraction and when indicated, for the creation of the anastomosis. Gastrointestinal continuity is finally restored by a stapled or hand-sewn intrathoracic esophagogastric anastomosis. A maximum of one thoracic drain is placed on each side.

### Paravertebral analgesia (intervention)

At the start of the thoracic phase of MIE, the surgeon places a paravertebral catheter at level T4–5 under thoracoscopic vision. After administering an initial bolus of 20 mL of bupivacaine 0.125%, continuous infusion of bupivacaine 0.125% is started and postoperatively continued at an infusion rate of 8–12 mL/hour, depending on the patient’s weight and comfort. Patient-controlled intravenous opioid analgesia is additionally provided according to the local protocol in each center.

### Epidural analgesia (control)

An epidural catheter is placed by the anesthesiologist at an intervertebral level between T5 and T8 prior to the induction of general anesthesia. After induction, a bolus of 5–10 mL of bupivacaine 0.25% is administered and traditional continuous epidural analgesia is initiated (bupivacaine 0.125% + sufentanil 0.5 mcg/ml) in a dose of 6–14 mL/hour, depending on the patient’s weight and comfort. Patient-controlled intravenous opioid analgesia is provided as escape medication according to the local protocol in each center.

### Outcome measures

The primary outcome measure is the total score on the Quality of Recovery 40 (QoR-40 [21]) questionnaire on the morning of postoperative day 3. The QoR-40 is a validated composite endpoint that can be used to evaluate analgesic modalities for postoperative pain control [22]. The main secondary outcome measures include the QoR-40 scores on postoperative days 1–2, the patient’s perception of postoperative pain management on days 1–3 (International Pain Outcomes (IPO) questionnaire), the need for escape pain medication on days 1–3, additional opioid consumption on days 1–3, technical complications, analgesia related side-effects, need for inotropic and vasopressive medication, length of stay on a monitored unit and in the hospital, postoperative complications, quality of life at 3 and 6 months after surgery, pain at 3 and 6 months after surgery (VAS score), and cost-effectiveness.

### Data collection

**Fig. 2:**
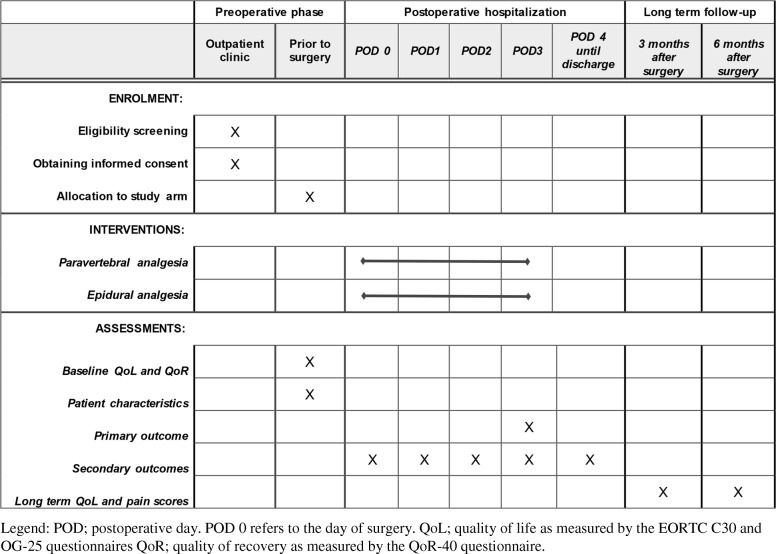
PEPMEN timeline of study procedures

All data will be collected and stored in Castor EDC (https://www.castoredc.com) and the coordinating investigators will oversee the overall data collection process. Castor EDC generates a subject number for each patient and securely stores all entered research data in a pseudonymized fashion. A code file that links subjects numbers to individual patients will be securely stored in each center, and will only be accessible to authorized study staff. Collected baseline data (age, gender, body mass index, comorbidities), treatment details (neoadjuvant therapy, surgical techniques, postoperative complications, mortality), and data regarding blood pressure (i.e. vasopressor and inotropic use, fluid administration, weight) will be prospectively entered in a case report form with built-in validation checks. Patient-reported outcome measures (PROMs) will be collected during the postoperative hospitalization by asking patients to complete the QoR-40 and IPO questionnaires on postoperative days 1, 2, and 3. Furthermore, Quality of life questionnaires (EORTC QLQ-C30 and QLQ-OG25) will be sent to patients before surgery, at 3 months follow-up, and at 6 months follow-up after esophagectomy. Pain scores (VAS) at 3 and 6 months after esophagectomy will be collected and registered during the patients’ regular outpatient follow-up visits. The timeline of the study procedures is summarized in Fig. 2.

### Quality control

Data collection and management are centrally monitored in all centers. The independent monitor of the study will visit each participating center at the start of inclusion, after 1 year of inclusion, and at the end of inclusion. All paravertebral procedures performed during the study will be recorded and stored for quality control. Serious adverse events related to the study procedures will be reported to the sponsor of the study without any undue delay.

### Sample size

The QoR-40 questionnaire was used as the primary outcome measure to calculate a sample size based on superiority of paravertebral analgesia when compared to epidural analgesia [21]. Backward and forward translations of the QoR-40 were validated by the study consortium to ensure the accuracy of the Dutch version. Based on previous literature, a QoR-40 score of 182 points with a standard deviation of 14 on postoperative day 3 is expected in the control group [23]. A difference of 6 points on the QoR-40 was assumed to indicate a minimally clinically relevant difference based on literature [24]. Hence, a total sample size of 172 patients is required (86 patients per group) to detect this difference with a statistical power of 80% and a significance level of (alpha) *P* < 0.05. Anticipating a 10% loss to follow up, 192 patients will be included. Only patients who withdraw before surgery will be replaced by new subjects.

### Statistical analyses

The difference in primary outcome between the two groups will be compared using an independent t-test based on the intention-to-treat principle. The primary outcome will be analyzed with a general linear model for continuous outcomes. In the analysis, the preoperative (baseline) QoR-40 measurement, age, and comorbidities will be included as potential confounders. Validity of the model (normality, homoscedasticity) will be assessed by means of residue analysis. A per-protocol analysis will additionally be performed in the same manner. Analyses of the secondary outcomes are performed depending on data type and distribution. Chi-square tests will be performed for categorical data. The t-test or Mann-Whitney U test will be used to compare continuous outcomes with a normal or non-normal distribution, respectively.

### Time schedule

The study has started on December 3rd 2019. The first 24 months will be used for patient inclusion. After finishing patient inclusion, there will be 6 months of follow-up and an additional 6 months of data analysis.

## Discussion

Thoracic epidural analgesia has long been the gold standard for pain management following esophagectomy. However, paravertebral analgesia is increasingly suggested as a good alternative for patients undergoing MIE, as the catheter can be placed under direct thoracoscopic vision possibly reduces the incidence of hypotensive events. Furthermore, by avoiding epidural catheterization and its potentially severe neurological complications, paravertebral analgesia may be safer. However, the currently available studies mainly included patients undergoing other thoracic surgical procedures and high-quality prospective studies investigating paravertebral analgesia for esophageal cancer surgery are lacking. Therefore, the PEPMEN study aims to compare paravertebral analgesia versus epidural analgesia in patients undergoing MIE by means of a prospective multicenter randomized controlled trial.

Adequate analgesia is one of the key parts of enhanced recovery after surgery (ERAS) protocols for major surgical procedures, aiming to achieve effective pain control whilst avoiding the side-effects of high doses of systemic opioids. These aims were also described in the recently developed ERAS Society guidelines for esophagectomy, which state that regional analgesia should be the backbone of pain management in enhanced recovery after esophagectomy protocols [25]. In absence of trials in patients undergoing MIE, both epidural and paravertebral analgesia were considered to be suitable options based on extrapolated evidence, although no recommendations in favour of either modality could be made [25]. Retrospective cases series suggest that paravertebral analgesia is associated with less technical failure and reduces the incidence of hypotensive events, which may promote postoperative recovery [26, 27]. Combined with inconclusive ERAS recommendations regarding the use of either epidural or paravertebral analgesia, these retrospective findings represent an important rationale to perform a prospective randomized controlled trial comparing paravertebral and epidural analgesia regarding perioperative outcomes and treatment costs in patients undergoing MIE.

In contrast to epidural analgesia, paravertebral analgesia can only induce a unilateral sensory block of thoracic dermatomes. Nonetheless, this unilateral thoracic block is expected to achieve adequate control of chest pain after MIE, especially when combined with patient-controlled intravenous opioid analgesia as an escape for breakthrough discomfort [28]. If paravertebral analgesia in fact achieves adequate pain control, the avoidance of epidural-related side-effects likely facilitates fast recovery after MIE [29, 30], which is the primary interest of this study. Since postoperative recovery is multifactorial, a composite endpoint was considered to be most appropriate and therefore the QoR-40 questionnaire was chosen as primary outcome. The QoR-40 consists of 40 questions that are divided in separate sections that aim to evaluate the presence and extent of pain, symptoms, comfort, emotional well-being, physical independence, and satisfaction with treatment [21]. As the hypothesized advantages of paravertebral analgesia are expected to impact several of these sections, the composite QoR-40 score is considered to be a good parameter for postoperative recovery.

One might argue that the current randomized study design could be replaced by a prospective cohort study with sequential measurement of a control group followed by an intervention group which might be acceptable regarding the risk of selection bias, as the demographics of esophageal cancer patients are not expected to change within a period of a few years. However, it should be noted that the intraoperative techniques and perioperative facets of enhanced recovery protocols are continuously subjected to alterations based on new developments in the field of MIE. In case of a non-randomized sequential prospective design, changes in parts of the perioperative protocol during the study (e.g. the postoperative mobilization program) would not be possible without potentially inflicting a bias that significantly impacts the interpretability of the results. By choosing a randomized study design with stratification per center, the participating centers can still implement perioperative developments in other areas than the pain management regimen without compromising the reliability of the comparison between analgesic techniques.

In summary, the PEPMEN study is a randomized controlled multicenter trial comparing paravertebral versus epidural analgesia in patients undergoing MIE in 4 high-volume centers for esophageal cancer surgery in the Netherlands. The primary endpoint will be the quality of recovery, as measured by the QoR-40 questionnaire on postoperative day 3. By improving the quality of recovery and shortening the length of stay on the ICU, paravertebral analgesia is expected to reduce the costs of perioperative care for patients undergoing MIE. A total sample size of 192 patients is required and the duration of the study will be 3 years.

## Data Availability

The datasets of this study will be available from the corresponding author on reasonable request after completion.

## Abbreviations

ASA: American Society of Anesthesiologists score
DMC: Data Monitoring Committee
eGFR: Estimated Glomerular Filtration Rate
ERAS: Enhanced Recovery After Surgery
IPO: International Pain Outcomes questionnaire
MIE: Minimally Invasive Esophagectomy
PROMs: Patient Reported Outcome Measures
QoR-40: Quality of Recovery 40 questionnaire
VAS: Visual Analog Scale
